# Pleiotropic Devitalization of Renal Cancer Cells by Non-Invasive Physical Plasma: Characterization of Molecular and Cellular Efficacy

**DOI:** 10.3390/cancers15020481

**Published:** 2023-01-12

**Authors:** Andreas Nitsch, Caroline Sander, Benedikt Eggers, Martin Weiss, Eva Egger, Franz-Josef Kramer, Holger H. H. Erb, Alexander Mustea, Matthias B. Stope

**Affiliations:** 1 Department of Trauma, Reconstructive Surgery and Rehabilitation Medicine, University Medicine Greifswald, Ferdinand-Sauerbruch-Straße, 17475 Greifswald, Germany; 2 Department of Gynecology and Gynecological Oncology, University Hospital Bonn, Venusberg-Campus 1, 53127 Bonn, Germany; 3 Department of Oral, Maxillofacial and Plastic Surgery, University Hospital Bonn, Welschnonnenstr. 17, 53111 Bonn, Germany; 4 Department of Women’s Health, Eberhard Karls Universität Tübingen, Calwerstraße 7, 72076 Tübingen, Germany; 5 Department of Urology, Technische Universität Dresden, Fetscherstraße 74, 01307 Dresden, Germany

**Keywords:** renal cell carcinoma, cold atmospheric plasma, non-invasive physical plasma, apoptosis, membrane permeability

## Abstract

**Simple Summary:**

Renal cell carcinoma (RCC) has an extremely poor prognosis, as there are few therapeutic options. In addition, resistance often develops during the administration of cytostatic drugs, preventing further drug-based therapy. New therapeutic modalities to reduce tumor cells would therefore be an important advance in the cancer therapy of RCC. In the present research study, non-invasive physical plasma (NIPP) was used to treat RCC cells. It was found that both cell growth and cell motility of RCC cells could be significantly reduced. Furthermore, programmed cell death was induced, so that some of the cancer cells actually died. Cellular analysis of NIPP treated cancer cells indicated that exposure to NIPP resulted in increased permeability of the cytoplasmic membrane to low molecular weight substances. This may contribute to the inhibition of the physiological activities of the cancer cells. The present results suggest that treatment with NIPP may represent a promising, innovative, and non-chemical option for the therapy of RCC and cancer in general. In combination with established therapeutic modalities, therapeutic effects would be enhanced and resistance would be minimized by the simultaneous application of multiple modes of action.

**Abstract:**

Renal cell carcinoma (RCC) is the third most common urological tumor and has an extremely poor prognosis after metastasis has occurred. Therapeutic options are highly restricted, primarily due to resistance to classical chemotherapeutics. The development of new, innovative therapeutic procedures is thus of great urgency. In the present study, the influence of non-invasive physical plasma (NIPP) on malignant and non-malignant renal cells is characterized. The biological efficacy of NIPP has been demonstrated in malignant renal cell lines (786-O, Caki-1) and non-malignant primary human renal epithelial cells (HREpC). The cell responses that were experimentally examined were cell growth (cell number determination, calculation of growth rate and doubling time), cell motility (scratch assay, invasiveness assay), membrane integrity (uptake of fluorescent dye, ATP release), and induction of apoptosis (TUNEL assay, caspase-3/7 assay, comet assay). A single NIPP treatment of the malignant cells significantly inhibited cell proliferation, invasiveness, and metastasis. This treatment has been attributed to the disruption of membrane functionality and the induction of apoptotic mechanisms. Comparison of NIPP sensitivity of malignant 786-O and Caki-1 cells with non-malignant HREpC cells showed significant differences. Our results suggest that renal cancer cells are significantly more sensitive to NIPP than non-malignant renal cells. Treatment with NIPP could represent a promising innovative option for the therapy of RCC and might supplement established treatment procedures. Of high clinical relevance would be the chemo-sensitizing properties of NIPP, which could potentially allow a combination of NIPP treatment with low-dose chemotherapy.

## 1. Introduction

Renal cell carcinoma (RCC) is the third most common (17.4 new cases per 100,000 inhabitants) but a fatal urological malignancy [1,2,3,4]. Men are affected more than twice as often as women (25.1 to 11.4 per 100,000) [1]. RCC accounts for 85% of all tumor diseases of the kidney, mostly affecting elderly individuals [1,2,3,4,5]. The mean age of diagnosis is 68 years for men and 71 years for women [5,6].

Tobacco consumption, obesity, and hypertension are among the most prominent factors for RCC development [7]. Despite novel abdominal imaging procedures, more than 25% of RCC patients are initially diagnosed with metastases [8].

The current therapy concepts depend on the tumor stage and the patient’s health condition. Focal therapies, e.g., cryofrequency ablation, are used to treat RCC patients with pronounced comorbidities or limited life expectancy [9,10,11,12]. In curative therapy, surgical resection of the tumor plays a pivotal role [13]. Kidney-preserving operations and partial nephrectomy can be performed on small, localized tumors [9,12,13]. Total nephrectomy in the palliative situation facilitates the reduction in the tumor burden [14,15]. 

Systemic antitumor therapy of RCC is only available to a very limited extent since there are frequently pronounced resistances to classical chemotherapeutic agents [16,17] and a lack of responsiveness to immunotherapeutic agents such as interferon-α or interleukin-2 [18,19]. The currently available targeted therapy approaches with tyrosine kinase and mTOR inhibitors are significantly more efficient and have thus replaced the classic therapy concepts mentioned above [9]. Targeted therapy prolongs overall survival from 21.8 to 26.4 months [20,21] with a 5 year survival rate of about 75%, depending on the tumor stage [5,22].

As with almost all malignancies, the molecular heterogeneity of RCC makes effective therapy difficult [23,24,25,26]. New therapeutic strategies are therefore urgently needed to improve the current treatment options. One option may be treatment with non-thermal physical plasma at atmospheric pressure (non-invasive physical plasma: NIPP) [27,28]. Various studies have shown the antiproliferative effect of NIPP on cancer cells of melanoma, breast, ovary, and lung, as well as skeletal sarcoma [29,30,31,32,33,34,35]. The antiproliferative effect of NIPP is based on the induction of apoptosis due to the production of reactive oxygen (ROS) and nitrogen species (RNS) [36] and limitations of cellular metabolism and membrane functionality [35,37,38]. The combination of NIPP treatment with classical chemotherapeutic agents also appears promising and could overcome existing chemoresistance [39]. To our best knowledge, the present investigation represents the first study on antitumoral effects of NIPP on RCC cells

## 2. Results

### 2.1. NIPP Treatment Inhibits Cell Proliferation by Reducing Cell Number and Growth Rate

**Figure 1:**
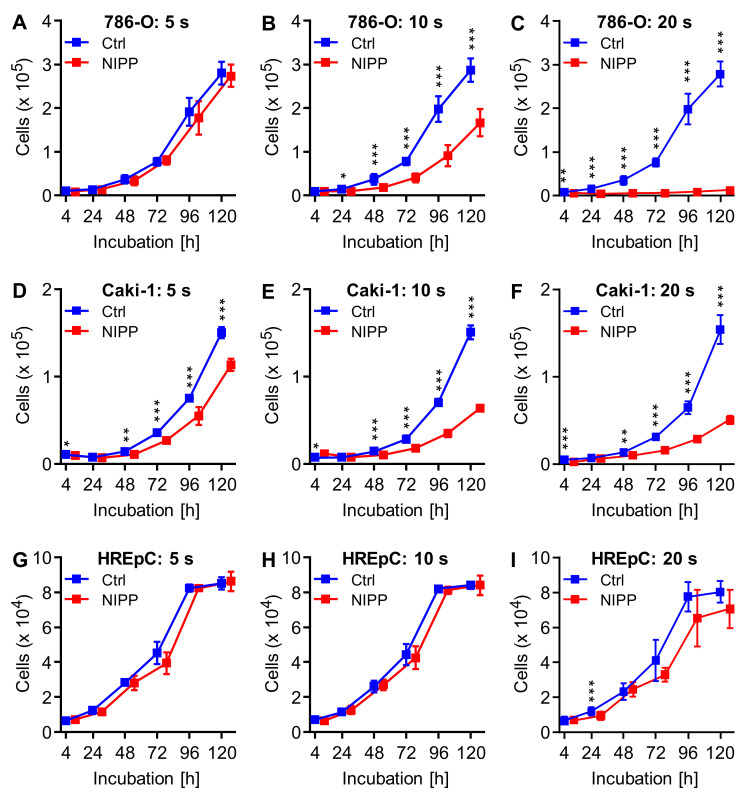
NIPP treatment reduces cell proliferation. The human renal cancer cell lines 786-O (**A**–**C**), Caki-1 (**D**–**F**) and the non-malignant HREpC cells (**G**–**I**), were treated with non-invasive physical plasma (NIPP) or the carrier gas argon (Ctrl). The number of living cells was determined at the given times using the CASY Cell Counter and Analyzer and were shown as mean ±SD. Differences between NIPP-treated cells and control cells were tested for significance using the paired *t*-test (* *p* < 0.05, ** *p* ≤ 0.01, *** *p* ≤ 0.001). For a better overview, the values of the NIPP treatment were shown slightly shifted to the right.

The malignant cell lines demonstrated inhibition of cellular growth after NIPP treatment (Figure 1). While in Caki-1 cells, a 5 s treatment resulted in a significant reduction in the cell number, only slight differences were observed in 786-O cells. From treatment times of 10 s, however, the proliferation of the malignant cell lines was significantly inhibited and reduced the number of living cells to about half after 120 h of incubation. Finally, a 20 s treatment led to almost complete growth inhibition in malignant cell lines. In contrast, the growth of the non-malignant cells was not significantly inhibited. However, it was observed that the primary cells (HREpC) began to die after 96 h regardless of treatment. Biostatistical analyses showed that these effects were due to the reduction in the number of cells during treatment and to a reduction in the growth rate of surviving cells (Table 1).

The calculated cell numbers of 786-O cells immediately after treatment differed significantly after NIPP treatments over 10 s and 20 s compared to controls (each *p* < 0.001). A comparison of the growth rates of 786-O cells demonstrated that the growth rate of the 20 s NIPP-treated cells was significantly reduced (*p* < 0.001). In contrast, NIPP treatment over 5 s and 10 s tended to result in a discrete increase in growth rate. After NIPP treatment of Caki-1 cells over 10 s and 20 s, the calculated cell numbers at time t = 0 (each *p* < 0.001) and the growth rates (each *p* < 0.001) decreased significantly. The treatment of HREpC showed no significant effects.

**Figure 2:**
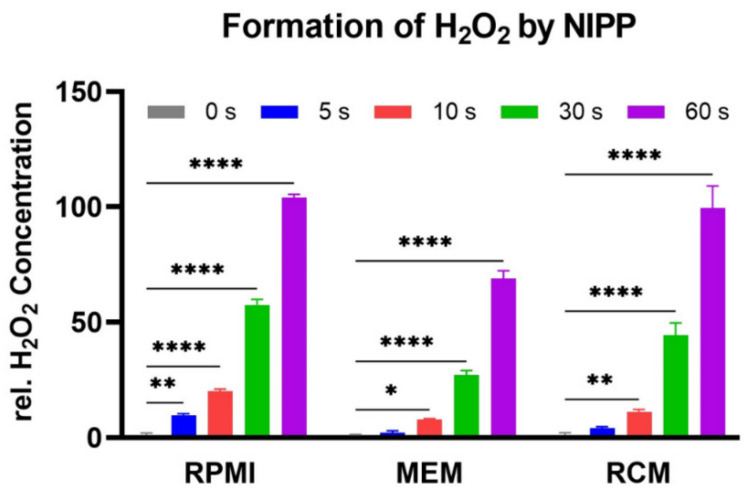
RPMI, MEM, and RCM cell culture media were treated with different NIPP treatment times and the formation of H_2_O_2_ was measured by Amplex Red assay. Data were expressed as mean ±SD. Significant differences between NIPP treatment and untreated controls (0 s; control = 1.0) are shown as follows: * *p* < 0.05, ** *p* < 0.001, **** *p* < 0.0001.

The generation of NIPP in atmospheric environment is associated with the formation of reactive oxygen species (ROS). To confirm this in the experimental setting, the cell culture media used, RPMI, MEM, and RCM, were treated with NIPP. The formation of H_2_O_2_ as a major representative of ROS was measured using an Amplex Red assay (Figure 2). The concentration of H_2_O_2_ formed increased with increasing NIPP exposure time in all cell culture media tested.

### 2.2. Incubation of Untreated Cells with NIPP-Treated Cell Culture Medium also Results in Cell Growth Inhibition

**Figure 3:**
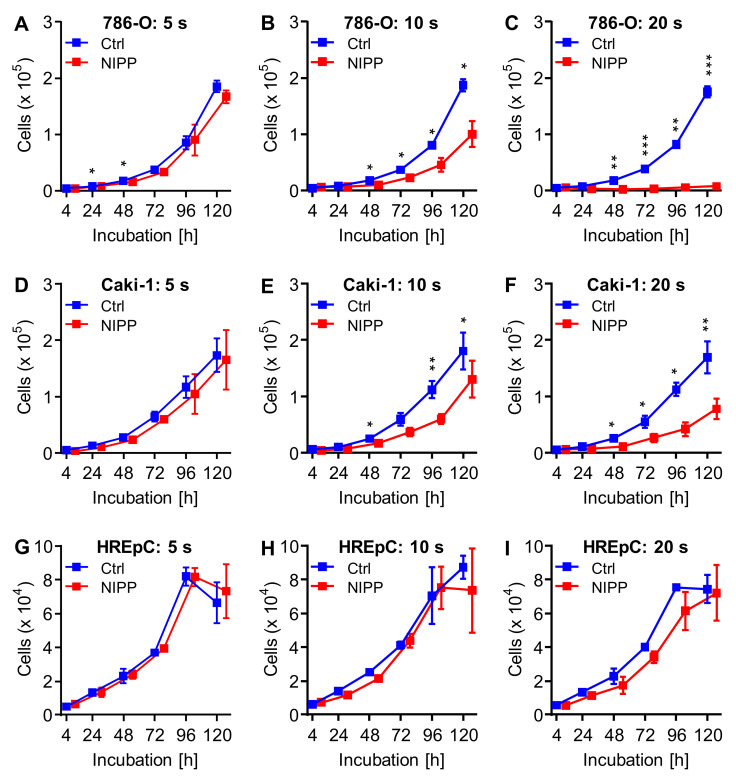
Treatment with NIPP-treated cell culture medium. The cell lines 786-O (**A**–**C**), Caki-1 (**D**–**F**) and non-malignant cells HREpC (**G**–**I**) were treated with non-invasive physical plasma (NIPP) activated cell culture media. After 24 h of pre-incubation, the cell culture medium was replaced by NIPP or argon-treated medium and the cells were incubated for further 120 h. The number of living cells was determined at the given timepoints by using the CASY Cell Counter and Analyzer and displayed as mean ±SD. Differences between the NIPP-activated media-treated cells and the control cells were tested for significance using the paired *t*-test (* *p <* 0.05, ** *p* ≤ 0.01, *** *p* ≤ 0.001). For clarity, the values of the NIPP treatment were shown slightly shifted to the right.

The experiments mentioned above demonstrated the growth inhibitory efficacy of NIPP treatment. We now examined whether NIPP effects can also be mediated indirectly through NIPP-treated cell culture medium and whether this also leads to reduced proliferation. For this purpose, the cells were seeded before treatment and pre-incubated 24 h. After preincubation, the cell culture medium was replaced by NIPP- or argon-treated medium and incubated for another 120 h. Treatment with a NIPP-activated medium inhibited cell proliferation in all cell lines (Figure 3).

Treatment with 5 s NIPP-activated medium resulted in a slight growth inhibition. Only 768-O cells showed significant differences at two points in incubation (24 h: *p* = 0.018, 48 h: *p* = 0.049). A 10 s treatment of cell culture medium resulted in approximately half-maximal inhibition in the malignant cell lines. Significant differences were found at all points from 48 h (*p* < 0.05). Cell proliferation was almost completely prevented in a NIPP-treated medium for more than 20 s. Here, significant differences were also found from 48 h on (*p* < 0.05). In contrast, the growth of the non-malignant cells (HREpC) was not significantly inhibited (Table 2).

Incubation of 786-O cells with NIPP-treated medium also led to a reduction in the number of cells. A significant difference was observed when incubated with the 20 s treated medium (*p* = 0.046). Indirect treatments of 786-O cells with NIPP activated medium for 10 s and 20 s led to a significant reduction in the growth rate (10 s: *p* = 0.004, 20 s: *p* < 0.001).

Indirect treatment of Caki-1 cells also reduced the number of cells at time t = 0. Significant differences were found at 10 s (*p* = 0.013) and 20 s (*p* = 0.012) treatment duration. For Caki-1 cells, the effects of indirect treatment were less pronounced. Here, there was a tendency for 5 s and 10 s NIPP treatment to increase the growth rate.

Indirect treatment of HREpC cells did not result in significantly different cell counts at time t = 0 or the growth rate. 

### 2.3. Treatment with NIPP Induces Apoptosis in Cells

**Figure 4:**
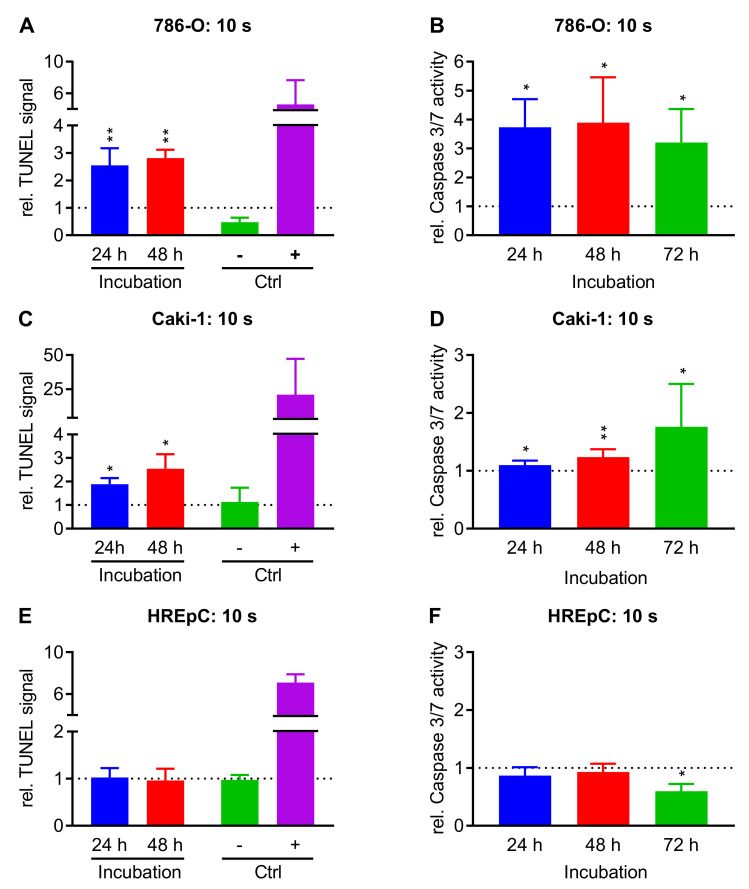
Apoptosis assays after NIPP treatment. Malignant cell lines 786-O (**A**,**B**), Caki-1 (**C**,**D**) and non-malignant HREpC (**E**,**F**) were treated with non-invasive physical plasma (NIPP) or carrier gas argon and examined for apoptosis using the established apoptosis detection methods TUNEL assay (**A**,**C**,**E**) and Caspase 3/7 activation assay (**B**,**D**,**F**). Means ±SD of the relative signal intensity are shown normalized to the respective argon control. The data were tested for significant differences using a paired *t*-test (* *p* < 0.05, ** *p* ≤ 0.01). To validate the TUNEL assay, positive and negative controls were carried out (+ nuclease-pretreated positive control,—unlabeled negative control). Those positive and negative controls were normalized to the mean of the argon controls.

Malignant cell lines 786 O, Caki-1, and non-malignant HREpC cells were used to examine whether treatment with NIPP leads to the activation of apoptotic processes. For this purpose, the established apoptosis detection methods TUNEL assay and Caspase 3/7 activation assay were performed. The cells were treated with NIPP or argon for 10 s. The TUNEL assay was performed 24 h and 48 h, the Caspase 3/7 assay 24 h, 48 h, and 72 h after NIPP treatment (Figure 4).

The treatment of 786-O cells with NIPP increased the relative TUNEL signal per cell to 2.6-fold (*p* = 0.005) after 24 h and to 2.8-fold (*p* = 0.003) after 48 h. At the same time, the relative caspase 3/7 activity increased by NIPP treatment (24 h: 3.7-fold, *p* = 0.031; 48 h: 3.8-fold, *p* = 0.037; 72 h: 3.2-fold, *p* = 0.025). Caki-1 cells exhibited similar results. A significant increase in relative TUNEL signal was observed 24 h (1.9.-fold, *p* = 0.044) and 48 h (2.5-fold, *p* = 0.046) after NIPP treatment. The relative caspase 3/7 activity was also increased (24 h: 1.1-fold, *p* = 0.026, 48 h: 1.2-fold, *p* = 0.006, 72 h: 1.8-fold, *p* = 0.047). Apoptotic effects in the non-malignant HREpC cells were less pronounced. NIPP treatment did not significantly increase the relative TUNEL signal at any of the observed time points. Caspase 3/7 activity was slightly reduced compared to the argon control. Seventy-two hours after NIPP treatment, the caspase 3/7 activity was significantly decreased compared to the control (0.6-fold *p* = 0.006).

### 2.4. Treatment with NIPP Reduces Cell Motility and Invasiveness

**Figure 5:**
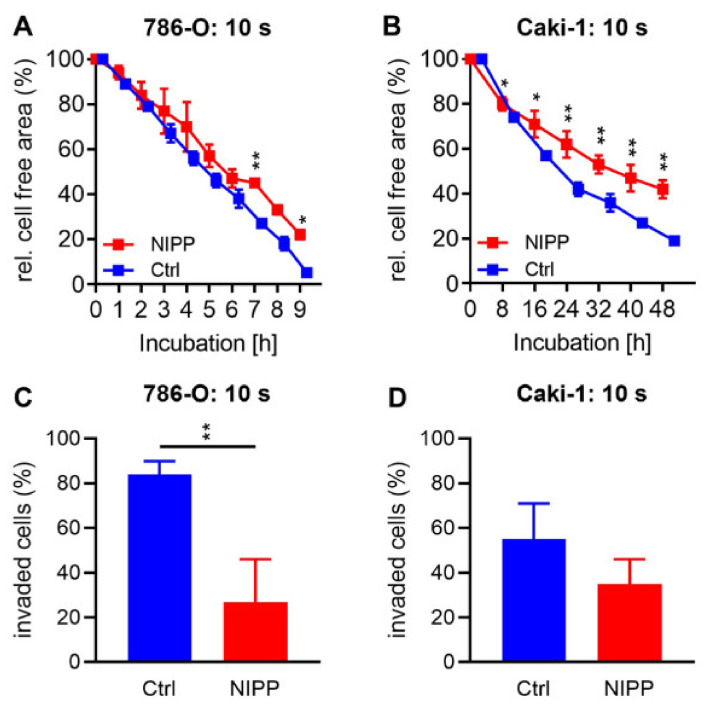
Motility assay and invasion assay after NIPP exposure. Cell lines 786-O (**A**,**C**) and Caki-1 (**B**,**D**) were treated with non-invasive physical plasma (NIPP) or the carrier gas argon (Ctrl). Scratch assay (**A**,**B**): A confluent cell layer was scratched and incubated in a live imagining microscope (Axio Observer Z1, Zeiss, Jena, Germany). Cells were cultured with low-serum conditions. The cell free area was photographed every half hour and measured at the indicated times. The relative area to the starting area at time t = 0 was evaluated. Migration assay (**C**,**D**): Treated cells were seeded in chambers with a semi-permeable membrane. An FCS gradient was established over the membrane. After 24 h of incubation, the cells on the top and bottom of the membrane were counted, and the invaded cells were compared to the total number of cells. Means ±SD are shown. Differences between the NIPP-treated cells and the control cells were tested for significance by students *t*-test (* *p <* 0.05, ** *p* ≤ 0.01).

Scratch assays were performed to investigate a potential impact of NIPP on cell motility. For this purpose, NIPP- and argon-treated cells were seeded and the confluent cell layer was scratched after 24 h. The cell layer was imaged hourly and the cell-free area was determined. An invasion assay was performed to explore the effect of NIPP on cell invasiveness. Cells were treated with NIPP and argon and seeded in chambers with a semi-permeable bottom membrane. An FCS gradient was established across the membrane and cells were incubated for 24 h. The adherent cells on the top and bottom of the membrane were counted (Figure 5).

Cell motility was cell line specific. The cell-free area of 786-O cells was almost completely closed after just a few hours. In contrast, Caki-1 cells could not completely close the free area for 48 h. The influence of motility through NIPP treatment also depended on the cell line. At any time of the evaluated measurement times, no significant differences were observed in 786-O cells. Significant differences, however, were found in Caki-1 cells after 8 h. HREpC cells died under low-serum conditions.

The cell lines also differed in their invasiveness. While more than 80% of the control-treated 786-O cells were invasive, only slightly than half of the Caki-1 cells were invasive. NIPP treatment significantly reduced the invasiveness of the 786-O cells. While 84% ±6% of the control-treated cells invaded through the membrane, the NIPP-treated cells were only 27% ±19% (*p* = 0.008). The NIPP impact was less obvious in Caki-1 cells; however, the results also indicated a reduction in the invasiveness in this cell line. While 55% ±16% migrated from the control cells, this proportion decreased to 35% ±11% (*p* = 0.149) after NIPP treatment. HREpC cells died under low-serum conditions.

### 2.5. Treatment with NIPP Impairs the Functionality of the Cytoplasmic Membrane

**Figure 6:**
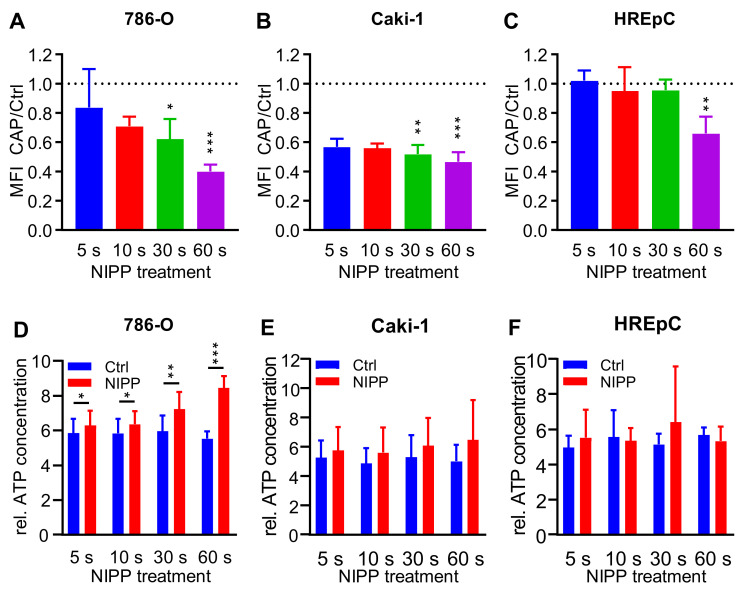
NIPP treatment induces loss of dye and ATP leak from cells Cell lines 786-O (**A**,**D**), Caki-1 (**B**,**E**) and non-malignant HREpC (**C**,**F**) were treated with non-invasive physical plasma (NIPP) or the carrier gas argon as a control. Loss of dye assay (**A**–**C**): Cells were stained with fluorescein diacetate and ethidium bromide. After 15 min of incubation, the cells were analyzed using a flow cytometer. The mean fluorescence intensity (MFI) of the living cells was evaluated. Normalized means + SD were shown. ATP release assay (**E**,**F**): After treatment cells were sedimented and extracellular ATP concentration in the cell-free supernatant was determined. Relative ATP concentrations + SD were shown. Differences between the NIPP-treated cells and the control cells were tested for significance with the paired *t*-test (* *p <* 0.05, ** *p* ≤ 0.01, *** *p* ≤ 0.001).

The generation of reactive species in the close environment and on the surface of the cells suggests that the efficacy of NIPP might alter the functionality of the cytoplasmic membrane. To investigate the integrity of the cytoplasmic membrane, cells were stained with FDA and treated with NIPP and argon. After an incubation of 15 min, cells were sedimented and the dye content of the cells was determined (Figure 6A–C). An ATP release assay was performed to investigate membrane permeability further. In contrast to the dye-based method, cells were not incubated with low-molecular-weight substances, but the release of an endogenously synthesized biomolecule was measured (Figure 6D–F). 

A reduced mean fluorescence intensity (MFI) was measured in all cell lines after NIPP treatment. In 786-O cells the intracellular FDA signal decreased significantly after 10 s treatment time (5 s: 0.83-fold, *p* < 0.240; 10 s: 0.71-fold, *p* = 0.002; 30 s: 0.62-fold, *p* = 0.009, 60 s: 0.40-fold, *p* < 0.001). For Caki-1 cells (5 s: 0.57-fold, *p* < 0.001; 10 s: 0.56-fold, *p* < 0.001; 30 s: 0.52-fold, *p* < 0.001; 60 s: 0.47-fold, *p* < 0.001) and HREpC cells (5 s: 1.02-fold, *p* = 0.596; 10 s: 0.94-fold, *p* = 0.0517; 30 s: 0.95-fold, *p* = 0.235; 60 s: 0.66-fold, *p* = 0.002). 

Measurements of ATP release could confirm data on FDA release. In 786-O cells, the NIPP treatment time of 5 s already resulted in a significant loss of ATP (5 s: 1.1-fold, *p* = 0.002, 10 s: 1.1 -fold, *p* = 0.023; 30 s: 1.2-fold, *p* = 0.002; 60 s: 1.5-fold, *p* = 0.002). Dose-dependent differences were also observed in Caki-1 cells; however, these were not significant at any of the treatment times investigated. HREpC cells displayed no significant differences between NIPP and control.

## 3. Discussion

NIPP’s cell growth-inhibitory effects have been proven in numerous studies with different types of malignant cells [38,40,41,42,43]. NIPP is a multi-component system consisting of ROS and other charged and uncharged particles, electric fields, and UV light. NIPP-derived ROS appear to play a central role as therapeutic pathways in the therapy of various malignancies [44,45]. It appears that the cell non-autonomous mechanisms manifest themselves biologically as a physical effect of the components of the NIPP. There is evidence that NIPP treatment of malignant cells leads to increased compaction of the cell membrane and thus to its biological protective function for the cell [35,46]. In addition, much is reported in the literature about the NIPP-induced apoptosis mechanisms stimulated in malignant cells [38,47,48]. 

In the field of uro-oncology, investigations on the application of NIPP in prostate and bladder tumors have already been carried out [49,50]. In our current study, the previously discussed effects of activation of apoptosis cascades and impairment of membrane permeability are demonstrated for the first time in malignant renal cells.

It was shown that a single NIPP treatment significantly inhibited cell growth of RCC cells. Furthermore, the strength of the antiproliferative effect increased with the duration of NIPP treatment, as was confirmed for other malignancies including ovarian, breast, and pancreatic cancer cells [32,38,51]. The primary noxious agent leading to this growth inhibition is reactive low-molecular-weight compounds, mainly reactive oxygen, and nitrogen species [37,52]. These are formed at the interfaces of NIPP and ambient atmosphere and of NIPP and liquid medium [53]. Therefore, the concentration of reactive species increases with continued treatment [36].

Reactive species as the predominant active component were also shown to inhibit cell growth not only in direct NIPP treatment. Growth inhibition also occurred after indirect treatment when untreated cells were incubated with NIPP-treated medium. In both cases, reactive species’ highly elevated extra- and intracellular concentrations lead to cell cycle arrest and cell death [54]. Conversely, low intracellular concentrations of reactive species activate the cell cycle and induce increased cell proliferation [54,55]. These effects are used for example in NIPP therapy of chronic wounds [56]. The main difference between direct and indirect NIPP treatment is that direct treatment may also have biological effects due to electromagnetic and thermal radiation. In contrast, indirect treatment only enables reactive species to be transported through the liquid medium [29,57]. Therefore, the cell biological effects of indirect treatment often appear less pronounced [58]; however, the present examinations have shown that NIPP treatment is almost equally effective for malignant and non-malignant renal cells, which was also observed in other studies [37,52]. In addition, the experimental use of NIPP can also lead to chemical changes in cell culture medium components in both direct and indirect treatment. This would also interfere with cellular metabolism, e.g., by chemically modifying amino acids, and thus cause a decrease in cell growth [36,59].

Since NIPP treatment did affect not only cell growth but also other central physiological functions of the cells, it had to be assumed that the NIPP effect cannot be solely attributed to disturbances in metabolic processes. It also seemed that general signal and effector cascades were dysregulated by NIPP components. Membrane permeability experiments for fluorescence-labelled low-molecular substances demonstrated an increased permeability after NIPP treatment. Cellular ATP molecules could also cross through the cytoplasmic membrane into the extracellular space, which may have contributed to metabolically induced growth inhibition. Comparable studies confirm this in melanoma and pancreatic carcinoma cells [60]. Immunological functions, however, cannot be excluded in this procedure. In apoptotic processes ATP may be secreted to activate macrophages [61,62].

Alterations in membrane functionality may also have contributed to NIPP-induced inhibition of cell motility. Membrane-bound adhesion proteins such as integrins and cadherins are central factors for cell contacts and cell mobility and be modulated by CPA treatment [56,63]. Treatment with NIPP can lead to chemical modifications of biomolecules, e.g. through oxidation processes. Therefore chemical modification of cellular surface factors such as lipids and proteins is not unlikely [64,65]. Inhibition of metastasis and invasiveness has already been shown for ovarian, colon, and hepatocellular carcinoma cells [32,66,67] and has also been demonstrated in RCC cells. The present study showed clear differences between the individual cell lines. The 786-O cells had a significantly higher metastatic and invasive potential than Caki-1 cells. The cell-free area in the scratch assay was closed six-fold faster by 786-O cells and the proportion of invasive cells was 80%, about one third higher than in the other two cell lines. This high cell mobility of 786-O cells was also demonstrated in other studies [68].

Another important cellular effect of NIPP treatment is the activation of the apoptotic machinery [69,70]. This has been shown at the activation level of the apoptotic proteases’ caspase-3 and caspase-7, as well as the apoptosis-specific degradation of genomic DNA [71] This difference was also reflected in the apoptosis assays. Here the non-malignant cells showed a lower and later apoptotic response. 

One hypothesis on the biological effect of NIPP suggests that cancer cells are significantly more affected by NIPP treatment than neighboring non-malignant cells [72]. This assumption is not uncontroversial and was also not supported by a recent study of us analyzing four cell lines after the treatment with three NIPP devices [73]. Although not the primary question, the present research was suitable for providing new insights into this hypothesis.

Cellular motility, invasiveness, and membrane permeability showed no significant differences between malignant and non-malignant cells after NIPP treatment. The question of a significantly stronger effect of NIPP on cancer cells still cannot be answered definitively. It is possible that permanent cell lines do not represent a suitable model system for these investigations due to their degenerated signaling networks and resulting unphysiological cell responses. The use of models with primary material might be able to remedy this situation.

The present studies on the biological effects of NIPP on renal cells show clear antiproliferative and antionogenic effects of NIPP treatment. These are mainly due to the induction of apoptosis and the disturbance of cytoplasmic membrane function. Treatment of tumors with NIPP remains a promising innovative option for new therapeutic methods in oncology. However, the effects of the different plasma devices with their different carrier gases must be adapted to the respective malignancy [37]. In the case of RCC, the combination of NIPP treatment and local chemotherapy could offer a promising new approach to treat this challenging malignancy.

## 4. Materials and Methods

### 4.1. Cell Culture

The human RCC cell lines 786-O and Caki-1 (both Cell Lines Service, Eppelheim, Germany) and the normal non-malignant renal cells HREpC (PromoCell, Heidelberg, Germany) were propagated at 37 °C and 5% CO_2_. For 768-O RPMI 1640 medium with 2 mM L-glutamine, 1% penicillin/streptomycin (P/S) and 10% fetal calf serum (FCS) was used. Caki-1 cells were propagated in minimal essential medium with 79.6 mg/L non-essential amino acids, 2 mM L-glutamine, 1 mM sodium pyruvate, 1% P/S, and 10% FCS (all reagents from PAN Biotech, Aidenbach, Germany). HREpC were cultured in renal epithelial cell growth medium 2 (RCM; Basal Medium and SupplementMix) obtained from (PromoCell, Heidelberg, Germany). 

### 4.2. Direct NIPP Treatment

A total of 1.0 × 10^4^ cells were suspended in 200 µL medium and transferred to a 24-well cell culture plate. NIPP treatment was performed with the kIN-Pen® MED plasma jet (Neoplas tools, Greifswald, Germany). The flow rate of the carrier gas argon was adjusted to 3 slm. Control cells were treated analogously, but without inflaming the plasma flame. Cell suspensions were treated for 5 s, 10 s and 20 s. Immediately after the treatment, 800 μL medium each were added to the wells and cells were incubated for 120 h. The number of living cells was determined after 4 h, 24 h, 48 h, 72 h, 96 h, 120 h using CASY cell counter and analyzer model TT (OLS OMNI Life Science, Bremen, Germany).

### 4.3. Indirect NIPP Treatment

A total of 5.0 × 10^3^ cells were seeded into the wells of a 24-well cell culture plate. After 24 h incubation, fresh preheated cell culture medium was activated with NIPP in another 24-well plate. Analogous to direct treatment, 200 μL of medium were treated per well. The treatment times corresponded to those of direct treatment. Immediately after treatment, the medium was carefully aspirated from the incubated cells and replaced by the treated medium. Afterwards, a further 800 µL of untreated medium was added to the wells. The cell number was determined and evaluated as described under Section 4.2.

### 4.4. Hydrogen Peroxide (H_2_O_2_) Formation Assay

A total of 200 μL of RPMI 1640, MEM (both containing 2 mM L-glutamine, 1% P/S and 10% FCS) and RCM (containing SupplementMix) were treated with NIPP for 0, 5, 10, 30 and 60 s. The cell culture medias were treated analogously to the cell suspensions in our other experiments. The treated media was diluted at 1:50 and the Amplex Red hydrogen peroxide assay (Thermo Fisher Scientific, Waltham, MA, USA) was carried out according to the manufacturer’s instructions. The background was subtracted. The data were analyzed relative to the control (control = 1.0).

### 4.5. Calculation of Growth Rate and the Nummber of Cell at the Time Immediatley after the Treatment

The growth rate and the number of cells at the time immediately after the treatment were calculated from the measured cell numbers after direct or indirect treatment using the following model:

Y = Y0 × exp(k × t)(1)

Y: cell count; Y0: cell count at t = 0; k: growth rate; t = time

For easier comparability, the doubling time was calculated from the growth rate using the following formula:

Doubling time = ln(2)/k(2)

Since the HNEpC began to die after 96 h, only the first 96 h after treatment were analyzed, in which the cells showed an exponential growth. 

### 4.6. TUNEL-Assay

A total of 5.0 × 10^4^ cells were treated with NIPP or argon for 10 s. 5.0 × 10^4^ (24 h incubation) or 2.5 × 10^4^ (48 h incubation) of the treated cells were transferred to a 96-well plate. Additionally, negative controls lacking fluorescent labelling and a nuclease-treated positive control were included. To be able to normalize the measured absorption to the cell number later, a second cell culture plate was simultaneously performed. The TiterTACS™ Colorimetric Apoptosis Detection Kit (Trevigen, Gaithersburg, MD, USA) was used according to the manufacturer’s instructions. Absorption was measured using the Infinite M200 plate reader (Tecan, Männedorf, Switzerland).

### 4.7. Caspase-3/7-Assay

A total of 5.0 × 10^4^ cells were treated with NIPP or argon for 10 s. 5 × 10^4^ (24 h incubation), 2.5 × 10^4^ (48 h incubation) or 1.25 × 10^4^ (72 h incubation) of the treated cells were incubated for 24 h, 48 h or 72 h. To be able to normalize the measured fluorescence intensity to the cell number, a second plate was planted out parallel. After the incubation period, the used medium was removed and 100 μL of Caspase 3/7 detection solution (CellEvent^TM^ Caspase 3/7 Green Detection Reagent (Thermo Fisher Scientific, Waltham, Massachusetts, USA) 2 µM in DPBS) were incubated for 45 min. The fluorescence (535 nm) was measured using a plate reader

### 4.8. Scratch-Assay

A total of 6.0 × 10^5^ (786-O, or 5.0 × 10^5^ (Caki-1) cells were seeded 24 h before the start of the assay. The confluent cell layer was scratched with a 250 µL pipette tip. Cells were washed twice with DPBS and 200 μL NIPP- or argon-treated medium wash to prevent the scratch from being closed by proliferation, the FCS concentrations in the medium were strongly reduced (786-O: 0.1% FCS, Caki-1: 0.5% FCS). By using the software Zen 2012 pro, the cell-free area was recorded over 48 h in intervals of 1 h. Evaluation of the cell-free area was determined with the ImageJ software using the MRI Wound Healing Tool plug-in. The measured cell-free areas were relativized to the cell-free area at the beginning of the experiment.

### 4.9. Invasion-Assay

**Figure 7:**
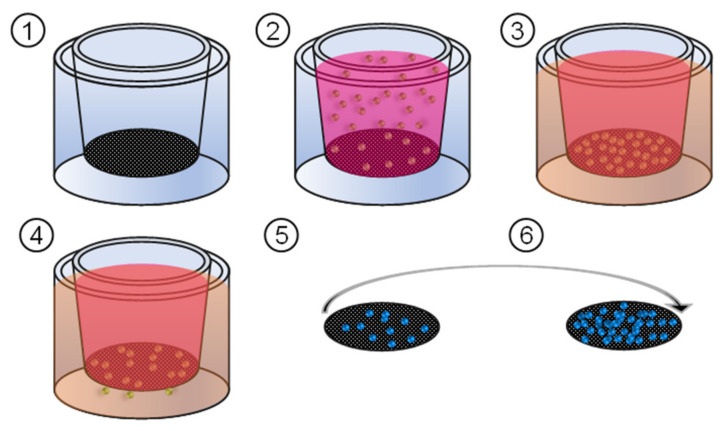
Scheme of the invasion assay (1) Cell culture inserts with an opaque bottom membrane were placed in the wells of a 24-well cell culture plate. (2) Cells, treated in FCS-free medium, were transferred to the inserts. (3) The well itself was filled with medium containing FCS. (4) Cells were able to follow the FCS gradient over 24 h and invade through the pores of the membrane. (5,6) Membranes were prepared and the cells were stained and counted.

To investigate the invasive capacity of the cells, invasion assays based on the Boyden chamber [74] were performed. For this purpose, cell culture inserts were inserted into the wells of a 24-well cell culture plate. The bottom of these inserts consisted of an opaque PET membrane with 8 µm pores. For the assay, 5.0 × 10^4^ cells were suspended in 200 μL FCS-free medium and treated with NIPP or argon for 10 s. Treated cell suspensions were filled with 500 µL FCS-free medium and transferred to the cell culture inserts. The wells of the cell culture plate itself were filled with FCS-containing medium. After 24 h incubation, the medium was carefully aspirated, and the inserts removed. The membranes were removed from the insert. After washing twice in DPBS the cells were stained with DAPI (500 nM in DPBS) and applied to slides. The membranes were covered with coverslips and signals were measured on both sides of the membrane. With the BZ-II Analyzer software, the number of cells was determined. The number of penetrated cells was evaluated in relation to the total number of cells. The test setup is shown in Figure 7. 

### 4.10. Loss of Dye Assay

**Figure 8:**
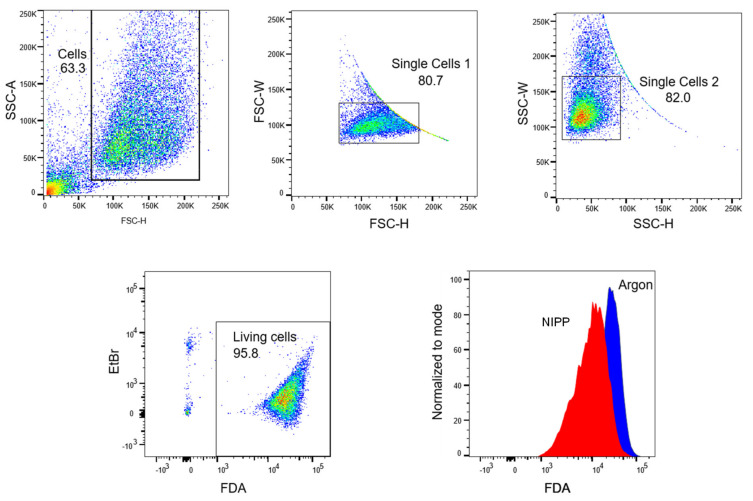
Gating strategy. Fluorescein diacetate (FDA) content per cell was analyzed by flow cytometry. Debris and doublets were excluded by forward- and side-scatter characteristics. Living cells were defined as ethidium bromide (EtBr)-negative and FDA-positive events. For analysis of data, the mean fluorescence intensity (MFI) of FDA was compared. SSC-A: side-scatter area, SSC-H: side-scatter height, FSC-W: forward-scatter width, FSC-H: forward-scatter height.

Cells were harvested and diluted to 1.0 × 10^6^ cells per milliliter in measuring buffer (Dulbecco’s phosphate-buffered saline (DPBS) with 10% FCS (*v*/*v*)). Subsequently, the cell suspension was treated with NIPP or argon for 60 s. Immediately after, the treatment cells were stained with 30 µg/mL ethidium bromide and 5 µg/mL FDA and incubated for 15 min on ice in the dark. After centrifugation, the labelled cells were resuspended in measuring buffer and analyzed in a FACSCanto flow cytometer (BD Biosciences, Heidelberg, Germany) with the FACSDiva 6.0 Software (BD Biosciences) and evaluated with FlowJo Software Version 10 (Tree Star Inc., Ashland, OR, USA). The gating strategy is shown in Figure 8. The signals of the NIPP-treated cells were normalized to the signals of control cells.

### 4.11. ATP Release Assay

Cells were harvested and adjusted to 1.0 × 10^6^ cells/ml with DPBS. A total of 200 μL of the cell suspension was treated with NIPPor argon for 5 s, 10 s, 30 s, or 60 s. After treatment, cells were incubated for 10 min at room temperature. After centrifugation, the relative ATP concentration of the cell-free supernatant was determined using the CellTiter-Glo 2.0 reagent (Promega GmbH, Walldorf, Germany) in an Infinite M200 plate reader.

## 5. Conclusions

We have shown that NIPP treatment effectively inhibits RCC cell proliferation. This effect was due to the impairment of cytoplasmic membrane and cell cycle functionality as well as the induction of apoptosis. These in vitro results give rise to the hope that NIPP treatment could also be used in vivo for the treatment of RCC including secondary tumors. If applicated intraoperatively, cells remaining in situ might be inhibited and their metastasis and invasiveness might be reduced. In the future, even endoscopic or laparoscopic NIPP application would be conceivable. An additional rinsing of the body cavity with NIPP-activated fluid, optionally in combination with low-dose chemotherapeutic agents, could further improve the prognosis. NIPP has the potential to significantly expand the opportunities for RCC therapy.

## Figures and Tables

**Table 1:** Calculated cell number at t = 0, growth rate and doubling time after direct NIPP treatment.

Cell	Treatment	Cells at t = 0		Growth Rate		Doubling Time (h)	
Line		Ctrl	NIPP	Ctrl	NIPP	Ctrl	NIPP
786-O	5 s	10,626 (±1074)	9866 (±1098)	0.02757 (±0.00089)	0.02789 (±0.00098)	25.1	24.8
	10 s	**10,892** ^1^ **(±1229)**	**5537** **(±698)**	0.02760 (±0.00010)	0.02907 (±0.00111)	25.1	23.8
	20 s	**9294** **(±757)**	**3518** **(±623)**	**0.02888** **(±0.00072)**	**0.00995** **(±0.00185)**	**24.0**	**69.6**
Caki-1	5 s	**4133** **(±278)**	**3094** **(±364)**	0.02995 (±0.00059)	0.02997 (±0.00104)	23.1	23.1
	10 s	**3001** **(±12)**	**3610** **(±413)**	**0.03253** **(±0.00062)**	**0.02380** **(±0.00103)**	**21.3**	**29.1**
	20 s	2975 (±160)	3173 (±268)	**0.03256** **(±0.00047)**	**0.02284** **(±0.00077)**	**21.3**	**30.4**
HREpC	5 s	7715 (±748)	6626 (±898)	0.02469 (±0.00111)	0.02612 (±0.00156)	28.1	26.5
	10 s	7079 (±645)	7154 (±753)	0.02553 (±0.00103)	0.02526 (±0.00119)	27.2	27.4
	20 s	6455 (±1267)	6333 (±1645)	0.02590 (±0.00222)	0.02419 (±0.002970)	26.8	28.7

^1^ Significant difference (*p* < 0.05) between non-invasive physical plasma (NIPP)-treated and the corresponding argon-treated control (Ctrl) were marked in bold.

**Table 2:** Calculated cell number at t = 0, growth rate and doubling time after treatment with NIPP activated media.

Cell	Treatment	Cells at t = 0		Growth Rate		Doubling Time (h)	
Line		Ctrl	NIPP	Ctrl	NIPP	Ctrl	NIPP
786-O	5 s	3850 ±127	3426 ±163	0.03203 ±0.00029	0.03205 ±0.00041	21.6	21.6
	10 s	3302 ±156	2679 ±329	**0.03365** **±0.00051**	**0.02888** **±0.00110**	**20.6**	**24.0**
	20 s	**3666** **±241**	**2766** **±621**	**0.03246** **±0.00059**	**0.00686** **±0.00251**	**21.4**	**101.1**
Caki-1	5 s	10,339 ±1629	7553 ±1830	0.22453 ±0.00146	0.02722 ±0.00221	28.3	25.5
	10 s	**8250** **±1169**	**3713** **±664**	0.02669 ±0.00129	0.02982 ±0.00168	26.0	23.3
	20 s	**8591** **±1222**	**3969** **±1250**	0.02585 ±0.00130	0.02481 ±0.00283	26.8	28.0
HREpC	5 s	5296 ±717	6117 ±660	0.02840 ±0.00151	0.02688 ±0.00121	24.4	25.8
	10 s	7964 ±1618	6813 ±1165	0.02275 ±0.00234	0.02511 ±0.00195	30.5	27.6
	20 s	6499 ±488	5518 ±1101	0.02551 ±0.00085	0.02512 ±0.00227	27.2	27.6

Significant difference (*p* < 0.05) between non-invasive physical plasma (NIPP) activated media-treated and the corresponding control (Ctrl) were marked in bold.

## Data Availability

The data can be shared upon request.
